# Long-term effectiveness of aripiprazole once monthly on functioning and quality of life in schizophrenia: results of year 2 of the ReLiAM study

**DOI:** 10.1186/s12888-024-06240-x

**Published:** 2024-11-14

**Authors:** Marc-André Roy, François Therrien, Matthieu Boucher, Oloruntoba Oluboka

**Affiliations:** 1 Centre de Recherche CERVO, Département de Psychiatrie Et de Neurosciences, Faculté de Médecine de L’Université Laval, 2525, Chemin de La Canardière, Porte A-1-2, Québec City, QC G1J 2G3 Canada; 2 Medical Affairs, Otsuka Canada Pharmaceutical Inc, St-Laurent, QC Canada; 3 Department of Pharmacology and Therapeutics, Faculty of Medicine and Health Sciences, McGill University, Montreal, QC Canada; 4 Department of Psychiatry, University of Calgary, Calgary, AB Canada

**Keywords:** Aripiprazole, Injectable, Monthly, Schizophrenia, Functioning

## Abstract

**Background:**

Aripiprazole once-monthly (AOM) has proven effective in the treatment of schizophrenia, although little is known about its impact on global functioning and quality of life beyond 1 year. Here, we investigate the continued impact of AOM on the participants of the ReLiAM study during the second year of follow-up.

**Methods:**

The participants who were evaluated at ≥ 1 time point during the second year of the ReLiAM study (months 15, 18, 21, and 24; year 1 completers) were assessed via the GAF scale. Secondary outcomes were reported on the SOFAS, CGI-S, and QLS.

**Results:**

109 (86%) completed at least 1 post-12-month visit and 33 (30.3%) patients completed the final assessment at month 24. The improvements observed in the year 1 completers in GAF total score were maintained through to year 2 completers. The improvements in CGI-S and SOFAS that were observed at the end of year 1 were also maintained through the end of the second year. Similar trends of sustained improvement in GAF total score, CGI-S score, and SOFAS were observed in the post-hoc analyses of the year 2 completers. Seventy-four percent (74.3%) of year 1 completers experienced mild treatment-emergent adverse events during the second year, the most frequently reported being weight gain, akathisia, and insomnia. Seventeen percent (17.4%) experienced serious adverse events. Similar findings regarding effectiveness and tolerability were reported in the year 1 completers and in year 2 completers.

**Conclusions:**

These findings suggest that the favorable effectiveness, including tolerability observed during the first year following AOM initiation, are maintained and may even continue to improve during the second year of treatment.

**Trial registration:**

ClinicalTrials.gov NCT02131415, first posted on May 6, 2014. Overall trial status: Terminated.

**Supplementary Information:**

The online version contains supplementary material available at 10.1186/s12888-024-06240-x.

## Introduction

Long-acting therapies (LAT), formulated as injectable medications, are as effective or more effective than their oral counterparts in managing the symptoms of schizophrenia [1]. Moreover, LATs offer a significant advantage over oral medications in reducing the risk of relapse, re-hospitalization, treatment nonadherence, and early mortality [2–6]. Although clinical guidelines support the use of LATs for the acute and maintenance treatment of schizophrenia, most patients are still offered oral medications for long-term disease control [7–13].

Aripiprazole is a dopamine and serotonin partial agonist/antagonist. Aripiprazole once-monthly (AOM) has been shown to reduce schizophrenia symptoms and proved superior in improving quality of life when compared to once-monthly dosing of paliperidone palmitate [14–16]. More recently, the Prevention of Relapse in Schizophrenia (PRELAPSE) study compared first hospitalization rates in patients receiving AOM or a clinician’s choice of treatment and observed that AOM delayed time to hospitalization in patients with schizophrenia [17].

The Real-Life Assessment of Abilify Maintena (ReLiAM) study was a Canadian naturalistic, non-interventional prospective cohort study designed to examine the effectiveness of AOM treatment on aspects of schizophrenia including symptomatology, functioning, and quality of life [18]. This study demonstrated that AOM improved the global functioning of patients with early (within 5 years of first diagnosis) and late-phase (longer than 5 years since first diagnosis) psychosis after 1 year. Despite growing evidence on the long-term utility of AOM, few studies have investigated the impact of AOM on schizophrenia functioning and symptoms for extended periods of time.

The primary objective of the current analysis is to assess the impact of AOM treatment on the global functioning (assessed with Global Assessment of Functioning [GAF] scale) of participants in the ReLiAM study after 2 years of treatment.

## Methods

### Study design and population

ReLiAM was a multi-site Canadian, non-interventional prospective cohort study in patients treated with AOM for schizophrenia for up to 24 months and was conducted between May 2014 and February 2017 [18]. A total of 250 patients were initially planned to be recruited across Canada. However, due to positive results reported in the interim analysis (after at least 50% of patients completed assessment at month 12), and a drop in the rate of patient enrollment, the final number of patients analyzed was 199. Of the 127 patients who were assessed at month 12 of ReLiAM, 109 (86%) continued with the study for at least 1 post-12-month visit (year 1 completers; Table 1). Here we report on treatment outcomes of patients who completed month 12 of study participation and were assessed at ≥ 1 time point during the second year of the study (months 15, 18, 21, and 24; identified as year 1 completers). A subset of the year 1 completers analyzed consisted of all patients who completed month 24 of study participation (identified as year 2 completers).

**Table 1:** Patients’ demographic and clinical characteristics

	**Year-1 completers (*n* = 109)**	**Year-2 completers** **(*n* = 33)**
**Age at consent** (years), mean ± s.d	33.5 ± 12.42	32.1 ± 12.92
**Gender**, n(%)		
Male	76 (69.7)	22 (66.7)
Female	33 (30.3)	11 (33.3)
**Race,** n(%)		
Caucasian	84 (77.1)	28 (66.7)
Black	14 (12.8)	4 (12.1)
Asian	5 (4.6)	0 (0.0)
Other	6 (5.5)	1 (3.0)
**Total GAF score at month 12**, mean ± s.d	60.8 ± 12.74	64.1 ± 12.26
**CGI-S score at month 12**, mean ± s.d	3.1 ± 0.96	3.0 ± 0.88
**BPRS total score at month 12**, mean ± s.d	40.3 ± 13.06	40.2 ± 11.05
**SOFAS total score at month 12**, mean ± s.d	52.6 ± 11.38	50.4 ± 11.62

*BPRS* Brief Psychiatric Rating Scale, *CGI-S* Clinical Global Impression-Severity scale, *GAF* Global Assessment of Functioning scale, *SOFAS* Social and Occupational Functioning Scale, *s.d.* standard deviation

All patients gave their signed, informed consent before participating in any study-related procedures and each site obtained research ethics approval from their local review boards.

### Assessments and outcome measures

The baseline assessment in the current report refers to the initial enrolment into the ReliAM study. The primary outcome measure of this study was the total score on the GAF scale through the second year of treatment. The Social and Occupational Functioning Assessment Scale (SOFAS), the Clinical Global Impression-Severity Scale (CGI-S), the abbreviated Quality of Life Scale (QLS), and the Brief Psychiatric Rating Scale (BPRS) were used as secondary outcome measures [19].

Furthermore, descriptive statistics on health care utilization (i.e., physician and emergency room visits and hospitalization) were recorded at each visit. Remission was defined as a score of 3 or less on 7 items of the BPRS (grandiosity, suspiciousness, unusual thought content, hallucinatory behavior, conceptual disorder, mannerisms, and blunted affect) for at least 6 months (i.e., 3 time points) after the assessment performed at the end of year 1 [20]. The rate of relapse was measured as the proportion of patients who achieved remission and who subsequently experienced worsening of symptoms that led to their hospitalization, or who had an increase of at least 1 point on the CGI-S from the last available measurement leading to a total score of 4 or more.

Safety signals in the form of adverse events (AEs), treatment-emergent adverse events (TEAEs), serious adverse events (SAEs), and changes in laboratory parameters were also recorded.

### Pharmacotherapy

All patients began treatment with 400 mg AOM, administered by their treating physician. In accordance with the recommendations in the product monograph, dose adjustments to 300 mg were made at the discretion of the investigators in special cases or in the event of adverse drug reactions. Treatment adherence was calculated at the end of the study and was defined as the number of injections, divided by the number of times of exposure to treatment in months, multiplied by 100. Patients also received additional care as per the treatment model of the individual clinics where they were treated. Adjunctive medications (including concomitant antipsychotics) were allowed.

### Statistical analyses

No imputations were performed for missing data. All analyses with the year 1 and year 2 completer datasets were observed case analyses.

Comparisons between assessment scores at baseline and at each time point were made with paired t-tests, and comparisons between early and late psychosis groups were analyzed by independent sample t-tests. All reported *p*-values are nominal and have not been corrected for multiple comparisons.

The reported data on year 1 and year 2 completers were not stratified into early- and late- phase schizophrenia and no post-hoc analysis was completed to distinguish the benefits of AOM for patients with schizophrenia at different phases of disease.

## Results

### Patient disposition

**Fig. 1:**
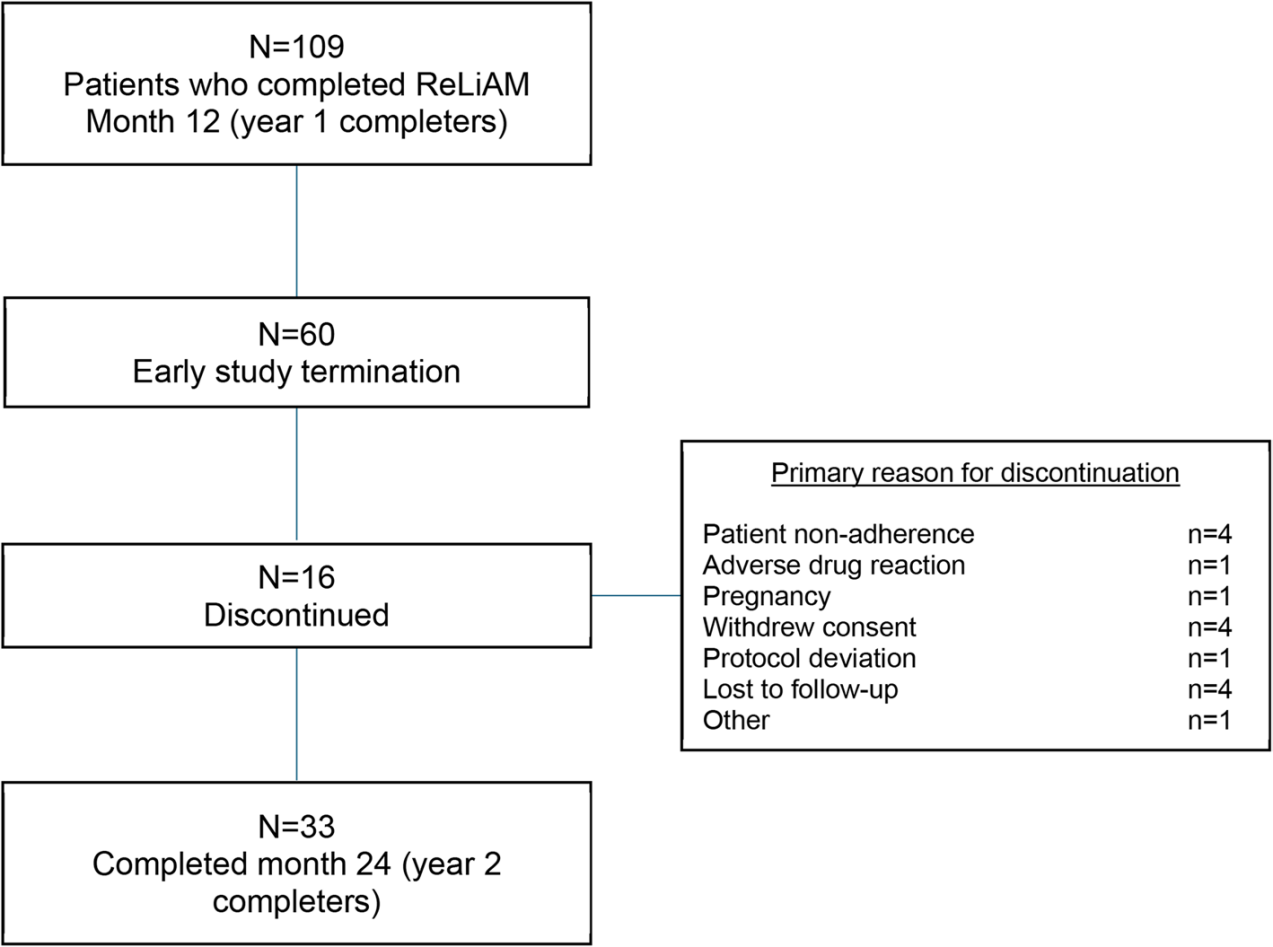
Patient disposition

As previously reported, 109 (86%) patients continued their participation in the ReLiAM study for at least 1 post-12-month visit (year 1 completers; Table 1). Among this group, 60 (60.6%) prematurely discontinued their participation in the study due to early study termination by the sponsor (Figure 1). Sixteen (14.7%) patients discontinued the study for reasons other than study termination, with the most common reasons being non-adherence, withdrawal of consent, and loss to follow-up (Figure 1). Importantly, 33 (30.3%) patients completed the final assessment at month 24 (year 2 completers: Figure 1).

The baseline demographic characteristics of the year 1 completers were similar to those of the patients who completed the month 24 assessments (year 2 completers). There were also no differences in mean scores of GAF total, SOFAS, CGI-S, QLS or BPRS at the beginning of the second year between the 1-year completers who did and did not complete the end of the 2-year treatment. (Table 1).

The mean dose of AOM at the beginning of the second year was 364.8 mg for year 1 completers and 368.2 mg for year 2 completers. Eight patients were prescribed a concomitant antipsychotic medication during year 2. Most patients received a monthly dose of 400 mg (72.5% of year 1 completers and 69.7% of year 2 completers). Four patients in the year 2 completer group, received dose adjustments for reasons other than AEs or breakthrough symptoms; no additional dose adjustments were made for those in the year 1 completers group.

### Effectiveness

Seventy-two patients (66.7%) in the year 1 completers group achieved remission and 21 (29.2%%) experienced a relapse during year 2 (Table 2). These proportions were similar for the year 2 completer subgroup of patients where 28 (84.8%) achieved remission and 7 (25%) relapsed.

**Table 2:** Remission and relapse on AOM during Year 2

	**Year-1 completers (*n* = 109)**	**Year-2 completers** **(*n* = 33)**
**Remission**^**a**^**, n (%)**	72 (66.7)	28 (84.8)
**Relapse**^**b,c**^**, n (%)**	21 (29.2)	7 (25.0)

^a^Remission was defined as a score of ≤ 3 on the following BPRS items for at ≥ 6 months, i.e., 3 consecutive visits: grandiosity, suspiciousness, unusual thought content, hallucinatory behavior, conceptual disorder, mannerisms, and blunted affect

^b^Relapse was assessed as the proportion of patients who achieved remission, who subsequently experienced (a) worsening of psychiatric symptoms that led to their hospitalization or withdrawal from the study, or; (b) an increase ≥ 1 point in the CGI-S compared to the last available measurement resulting in a score ≥ 4

^c^Percentages were calculated by taking “Yes” count of respective remission category as denominator

*BPRS *Brief Psychiatric Rating Scale*, CGI-S *Clinical Global Impression-Severity scale

**Fig. 2:**
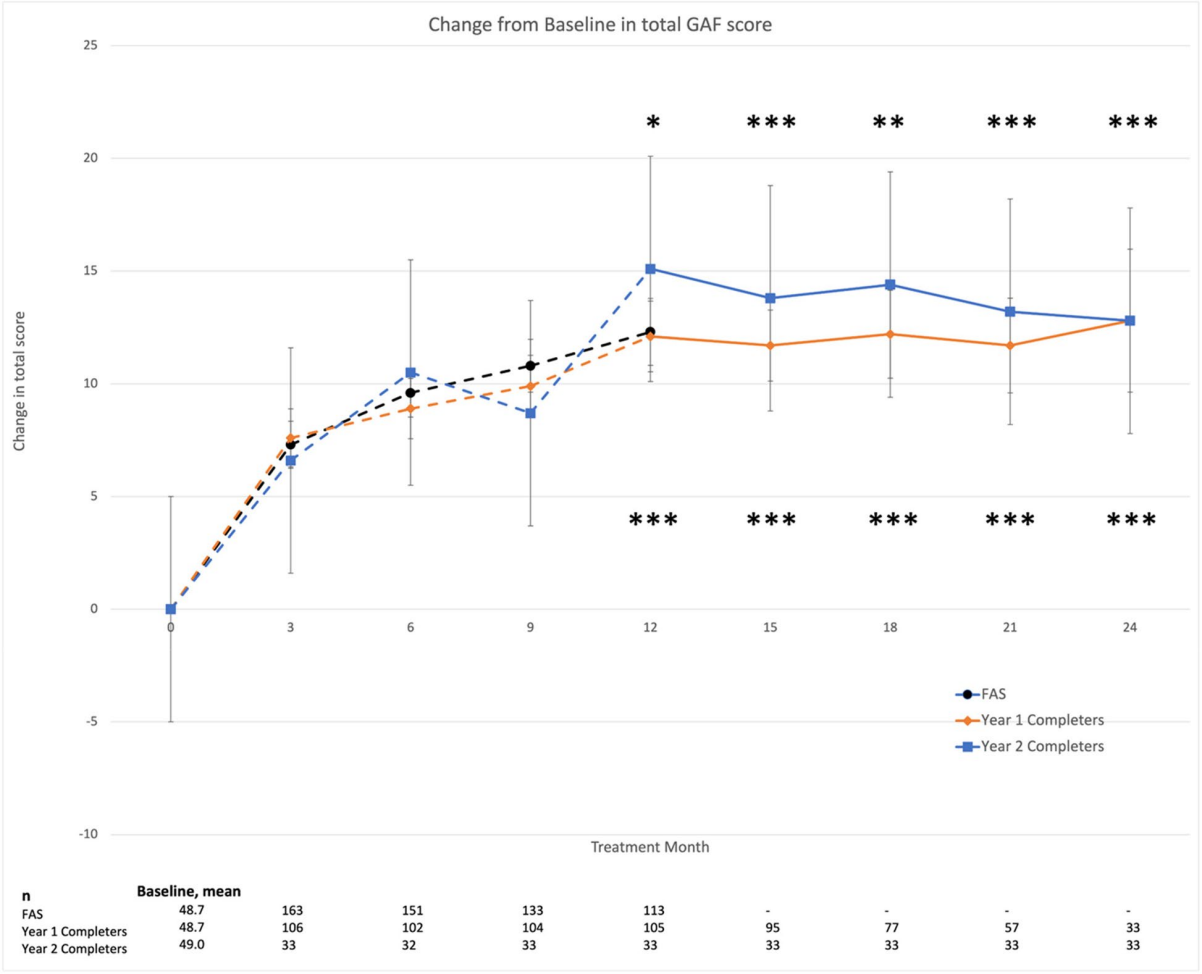
Change from baseline in GAF score. Data presented as mean ± SEM. Dotted lines represent the first year of the ReLiAM study and the solid lines represent the results of year 2 of the ReLiAM study; presented as year 1 completers in orange and the subcategory of year 2 completers in blue. *p < 0.05; **p < 0.01; ***p < 0.001 vs baseline defined as time of enrollment into the study. FAS, full analysis set; GAF, Global Assessment of Functioning scale; SEM, standard error of the mean

**Fig. 3:**
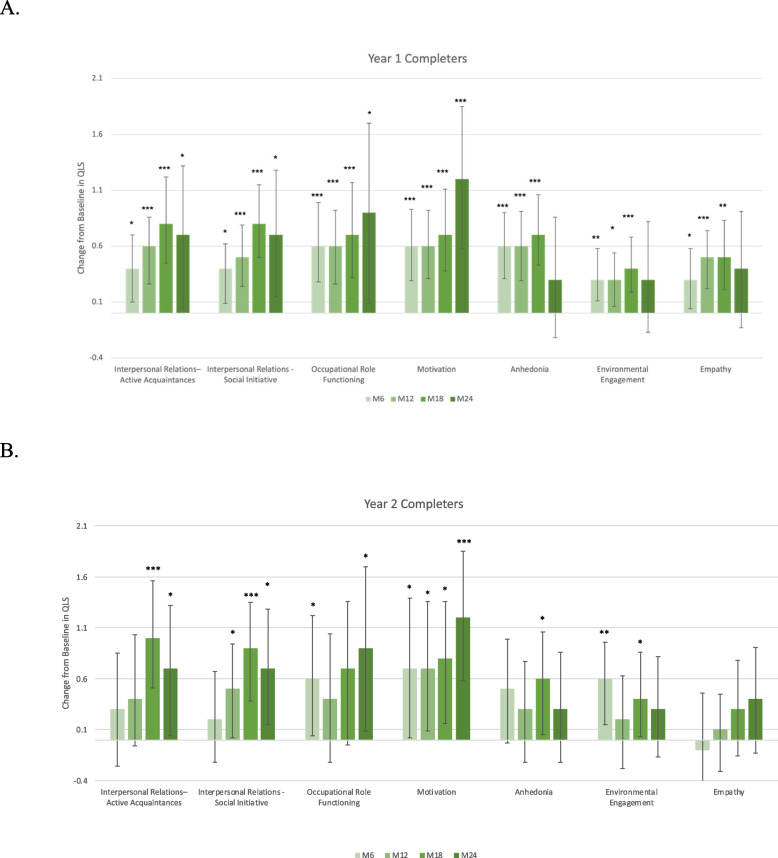
Change from baseline in Quality of Life Scale subscores by visit for (**A**) the year 1 completers and (**B**) year 2 completers. M, month. * *p* < 0.05, ** *p* < 0.01, *** *p* < 0.001 vs baseline defined as time of enrollment into the study

The previously observed increase in total GAF score at month 12 compared to baseline was 12.8 (18.2), reported as mean improvement (standard deviation [SD]), and was maintained through to month 24 for both year 1 and year 2 completers (Figure 2). Nominally significant improvements in change from baseline were observed beginning at 3 months through to 24 months in SOFAS and CGI-S for both year 1 and year 2 completers (Table 3). Improvements in total BPRS were also reported throughout (Table 3). Furthermore, improvements across all 7 QLS subcategories were observed up to month 18 in year 1 completers (Figure 3A). These changes remained nominally significant at month 24 in the subcategories of interpersonal relations (active acquaintances and social initiatives), occupational role functioning, and motivation (Figure 3A). No significant improvements were observed at month 24 in categories of anhedonia, environmental engagement, and empathy for year 1 completers (Figure 3A). Furthermore, no significant changes were observed between month 12 and month 24 on any of the effectiveness variables. A visual inspection of Figure 3 reveals a large SD, likely due to reduced sample size by month 24, as the numerical values remained similar to month 18 scores.

**Table 3:** Reported secondary outcome measures at each time point for year 1 and year 2 completers

Secondary Outcome Measure									
Month	**Baseline**	**3**	**6**	**9**	**12**	**15**	**18**	**21**	**24**
SOFAS									
Year 1 completers									
n	108	106	101	104	105	95	77	57	33
Mean ± s.d	50.4 ± 11.62	57.4 ± 11.41***	57.9 ± 11.37***	59.4 ± 12.31***	61.0 ± 12.86***	61.0 ± 13.41***	62.1 ± 12.73***	62.4 ± 12.93***	65.1 ± 11.57***
Year 2 completers									
n	33	33	31	33	33	33	33	33	33
Mean ± s.d	52.6 ± 11.38	58.0 ± 10.37**	60.8 ± 11.73***	60.7 ± 10.83***	64.1 ± 12.63***	63.7 ± 11.50***	63.9 ± 9.45***	64.1 ± 11.66***	65.1 ± 11.57***
CGI-S									
Year 1 completers									
n	108	105	100	103	106	93	74	54	32
Mean ± s.d	4.1 ± 0.78	3.5 ± 0.86***	3.3 ± 0.84***	3.2 ± 0.93***	3.1 ± 0.96***	3.1 ± 0.93***	3.0 ± 0.94***	3.0 ± 0.98***	2.9 ± 0.76***
Year 2 completers									
n	33	33	31	33	33	33	31	33	32
Mean ± s.d	3.8 ± 0.68	3.4 ± 0.66**	3.2 ± 0.73***	3.2 ± 0.80***	3.0 ± 0.88***	2.9 ± 0.70***	2.9 ± 0.81***	2.9 ± 0.86***	2.9 ± 0.76***
Total BPRS									
Year 1 completers									
n	108	106	102	104	105	95	77	57	33
Mean ± s.d	40.2 ± 11.05	33.3 ± 10.08	30.7 ± 7.80	29.3 ± 7.83	29.2 ± 8.27	28.7 ± 7.70	27.2 ± 7.89	26.9 ± 7.01	25.5 ± 5.28
Year 2 completers									
n	33	33	32	33	33	33	33	33	33
Mean ± s.d	40.3 ± 13.06	34.6 ± 11.91	31.5 ± 8.29	28.6 ± 6.96	30.2 ± 9.66	28.2 ± 6.21	26.5 ± 5.75	26.6 ± 5.62	25.5 ± 5.28

The total score for SOFAS ranges from 0 to 100. Reported. The CGI-S ranges from 1–7, where a score of 1 indicates no psychopathology and a score of 7 indicates severe psychopathology. The total BPRS ranges from 0–7: 0 = not assessed, 1 = not present, 2 = very mild, 3 = mild, 4 = moderate, 5 = moderately severe, 6 = severe, 7 = extremely severe. Total Score is calculated by adding 18 subscores for each subject at each visit. *P* value was calculated by using paired t-test of scores at each time point vs baseline defined as time of enrollment into the study. * *p* < 0.05, *** *p* < 0.001. *BPRS* Brief Psychiatric Rating Scale, *CGI-S* Clinical Global Impression-Severity scale, *SOFAS* Social and Occupational Functioning Scale

Similar improvements in GAF total score (Figure 2), CGI-S score, and SOFAS (Table 3) were observed in post-hoc analyses of the year 2 completers. Similar trends in overall improvements in QLS could be observed in year 2 completers (Figure 3B).

### Safety and tolerability

Although 74.3% (81/109) of patients in the year 1 completers and 84.8% (28/33) of year 2 completers experienced TEAEs, most were mild to moderate in severity and no TEAEs led to drug discontinuation in either group (Supplementary Table 1). Nineteen (17.4%) participants in the year 1 completers group reported SAEs, 17 of which led to prolonged hospitalization. Similar trends were observed for the year 2 completer subsets (Supplementary Table 1). The most common AEs in both groups were weight increase (17.4% in the year 1 completers and 15.2% in the year 2 completers), followed by akathisia in the year 1 completers (11.0%), and insomnia in the year 2 completers (12.1%). Mean (SD) weight at the end of Year 1 was 89.2 kg (21.7). Observed changes in mean weight during Year 2 were respectively +1.8 kg, +1.2 kg, +0.8 kg and -0.8 kg at Months 15, 18, 21, and 24. No deaths were reported during Year 2 of the study.

## Discussion

Although recent studies have demonstrated the efficacy of AOM in multi-cohort and subgroup analyses [21–25], the current study is the first to investigate the impact of AOM beyond 1 year of treatment. Here, using GAF as the primary measure, we report that the improvements observed during the first year were maintained at multiple time points, through the end of the year 2 period, compared to the year 1 assessment. This result was mirrored by improvements in all secondary measures (SOFAS, CGI-S, BPRS, and QLS). The benefits of AOM treatment in the current study were also evidenced by high remission rates for both year 1 and year 2 completers. A larger sample size would be required to fully assess clinical relapse with prolonged use of AOM; however, previous studies have shown that a longer treatment period prior to discontinuation does not reduce the risk of relapse [26]. The drug tolerability and incidence of AEs, SAEs, and TEAEs were similar to previous studies reporting on the use of AOM [17, 27].

Interestingly, the benefits of AOM treatment were previously reported to be higher in patients aged ≤ 35 years compared to those aged > 35 years [17, 28]. Based on these findings, an early treatment start may be beneficial for improvement of symptoms with the use of AOM. Future studies with larger sample sizes are required to investigate the clinical significance of these findings.

The findings of the present study must be interpreted considering the following limitations. First, due to the study termination following the pre-planned interim analyses, a high attrition rate was observed which may have biased the results. However, the fact that the 2-year completers were found to be very similar to the full cohort of 1-year completers makes this unlikely. Second, the modest sample size precluded performing the analyses for age-defined strata; therefore, it is not possible to determine whether the superior effectiveness observed in patients < 35 years of age in the QUALIFY study also apply to the present analysis. Third, the open-label and uncontrolled nature of the study inherently limit conclusions that could be drawn related to the comparative effects of the examined intervention. Finally, it should be acknowledged that results reported here were derived from observed data, hence from subjects successfully persisting in the study and likely deriving clinical benefits from the LAI administered.

In conclusion, the current study suggests that the benefits observed during the first year of AOM treatment are sustained over the second year, thereby further supporting the use of AOM in the long-term treatment planning for psychosis.

## Supplementary Information

Supplementary Material 1.

## Data Availability

For ethical reasons, to ensure the privacy of the patient-level data utilized in the current study, and for reasons related to data ownership by the sponsor, data cannot be made available. However, data could be made available upon request for the purpose of conducting meta-analytic review in future.

## Abbreviations

AE: Adverse Event
AOM: Aripiprazole Once-Monthly
BPRS: Brief Psychiatric Rating Scale
CGI-S: Clinical Global Impression-Severity Scale
FAS: Full Analysis Set
GAF: Global Assessment of Functioning
LAT: Long-Acting Therapy
PRELAPSE: Prevention of Relapse in Schizophrenia
QLS: Quality of Life Scale
ReLiAM: Real-Life Assessment of Abilify Maintena
SAE: Serious Adverse Event
SD: Standard Deviation
SEM: Standard Error of the Mean
SOFAS: Social and Occupational Functioning Assessment Scale
TEAE: Treatment-Emergent Adverse Event
