# Supplementary material for: Long-term effectiveness of aripiprazole once monthly on functioning and quality of life in schizophrenia: results of year 2 of the ReLiAM study
Source: BMC Psychiatry. 2024 Nov 14;24:797. doi: 10.1186/s12888-024-06240-x (PMC11562632; doi:10.1186/s12888-024-06240-x)
Supplement: Supplementary file 1 — Supplementary Material 1. [file 12888_2024_6240_MOESM1_ESM.docx]

|  | Year 1 completers (n=109) | Year 2 completers  (n=33) |
| --- | --- | --- |
| **Patients with any TEAE, n(%)** | 81 (74.3) | 28 (84.8) |
| **Patients with any SAE, n(%)** | 19 (17.4) | 3 (9.1) |
| **AE in ≥5% of patients in either dataset, n(%)** |  |  |
| Akathisia | 12 (11.0) | 3 (9.1) |
| Tremor | 7 (6.4) | 2 (6.1) |
| Insomnia | 6 (5.5) | 4 (12.1) |
| Anxiety | 5 (4.6) | 3 (9.1) |
| Psychotic disorder | 3 (2.8) | 2 (6.1) |
| Weight increased | 19 (17.4) | 5 (15.2) |
| Fatigue | 6 (5.5) | 1 (3.0) |
| Muscle rigidity | 4 (3.7) | 2 (6.1) |

**Supplemental Table 1**. Summary of adverse events. AE, adverse event; SAE, serious adverse event; TEAE, treatment-emergent adverse event.
